# Evaluation of changes in plasma levels of MMP-8, MMP-13, and Thrombospondin 2 in patients with Osgood–Schlatter disease (OSD)

**DOI:** 10.3389/fmolb.2025.1703728

**Published:** 2025-12-10

**Authors:** Monika Kulesza, Tomasz Guszczyn, Aleksandra Kicman, Rafał Marecki, Michał Stanisław Kicman, Sławomir Ławicki

**Affiliations:** 1 Medical University of Białystok, Department of Population Medicine and Lifestyle Diseases Prevention, The Faculty of Medicine, Białystok, Poland; 2 Medical University of Białystok, Department of Pediatric Orthopaedics and Traumatology, Białystok, Poland; 3 Medical University of Białystok, Department of Psychiatry, Białystok, Poland; 4 Independent Researcher, Białystok, Poland

**Keywords:** Osgood-Schlatter disease (OSD), matrix metalloproteinases, MMP-8, Mmp-13, thrombospondin 2, plasma

## Abstract

**Introduction:**

Osgood-Schlatter disease (OSD) is characterized by its relatively frequent occurrence and unknown pathomechanism. It mainly affects young athletes. Matrix metalloproteinases (MMPs) may be involved in the pathogenesis, while Thrombospondin 2 is involved in healing process. Purpose of this study was to determine changes in MMP-8, MMP-13 and Thrombospondin 2 levels in patients with OSD compared to control group (CG).

**Methods:**

The study was conducted on 140 patients with OSD (age range: 11–15), CG consisted of 100 individuals with minor hand injuries (age range: 12–15). Levels of MMPs and Thrombospondin 2 were determined in plasma using an immunoenzymatic method (ELISA).

**Results:**

Concentrations of MMP-13 (median: 496.5 ng/mL; p < 0.0001) and Thrombospondin 2 (median: 21.05 pg/mL; p < 0.0001) were higher in patients with OSD, while MMP-8 values (median: 26.60 ng/mL; p < 0.001) were lower in patients with OSD compared to CG (58.08 ng/mL; 14.43 pg/mL; 95.91 ng/mL; respectively). Significant correlations were found between the parameters studied, and the highest AUC (area under curve) was obtained for MMP-13 in the OSD group.

**Discussion:**

The studied compounds have potential as additional tests to distinguish OSD from other diseases, and MMP-13 may be involved in the pathogenesis of OSD.

## 1 Introduction

Osgood-Schlatter disease (OSD) is a common condition occurring during the maturation of the growth plate in physically active children and adolescents, especially those who engage in intensive training. OSD, also known as traction apophysitis or osteochondrosis, belongs to the group of sterile bone necrosis; it is an inflammation of the tibial tuberosity, just below the patella (Chandra et al., 2024; Preuss et al., 2024). It is estimated that the prevalence of OSD ranges from 9.8% to 12.9% of athletically active young people (Chandra et al., 2024). The time of onset of the disease is most often between the ages of 10 and 15 in boys and between 8 and 13 in girls. Boys are more commonly affected by the disease; however, the gender of the child does not affect the probability of developing the OSD (Chandra et al., 2024; Preuss et al., 2024; Guszczyn et al., 2024). The disease manifests as pain, swelling, and tenderness in the region of the tibial tuberosity that intensifies when running, jumping, squatting, or climbing stairs and subsides after rest (Chandra et al., 2024; Preuss et al., 2024; Guszczyn et al., 2024). Currently, the diagnosis of OSD is based on clinical history and imaging studies such as X-rays (Chandra et al., 2024; Preuss et al., 2024). According to our current knowledge, there are no scientific studies on the changes in biochemical parameters that are preserved in the course of OSD (Guszczyn et al., 2024). In general, OSD is a self-limiting disease, but the symptoms last about 12 months, and in exceptional cases can last up to 24 months. Therefore, about 90% of patients undergo conservative treatment to reduce discomfort associated with the disease, such as pain in the knee area and swelling. Conservative treatment, such as immobilizing the limb or applying cold compresses, significantly limits physical activity. Surgical treatment is reserved for children after the growth spurt (Guszczyn et al., 2024; Ladenhauf et al., 2020; Schultz et al., 2022).

The pathogenesis of OSD is currently poorly understood (Preuss et al., 2024; Guszczyn et al., 2024). Risk factors for OSD may include being overweight, increased tension and significant strength of the quadriceps muscle of the thigh, as well as decreased flexibility of the muscles of the posterior group of the thigh (Guszczyn et al., 2023; Smith and Varacallo, 2025; Indiran and Jagannathan, 2018). Chronic overload in the tibial tuberosity region due to excessive physical activity can cause multiple microinjuries that contribute to inflammation in the tibial tuberosity (Guszczyn et al., 2024). In addition, the pathomechanism behind the abnormal collagen metabolism in OSD is not understood. The disease is an example of sterile cartilaginous necrosis, in the development of necrotic foci, formed without the involvement of microbial agents. In addition, processes of increased degradation of the extracellular matrix are described in the course of OSD (Xu et al., 2017). The processes of increased matrix degradation are strongly influenced by biologically active compounds such as matrix metalloproteinases and Thrombospondin 2 (Kulesza et al., 2023).

Matrix metalloproteinases (MMPs) are proteolytic enzymes responsible for homeostasis of the extracellular matrix. The activity of these enzymes has been shown to be associated with the development of skeletal diseases such as vertebral degeneration, skeletal tumors, osteoporosis, and osteopenia. Therefore, it seems expedient to determine their potential role in OSD as well. In previous studies conducted by our team, we have shown that children with OSD have elevated or reduced blood levels of selected MMPs, indicating their potential involvement in the pathogenesis of this disease (Kulesza et al., 2023; Khalil, 2017).

Thrombospondin 2 is also an important regulator of the extracellular matrix. This protein has been shown to be involved in various processes related to the skeletal system, such as early fracture healing, bone growth, as well as bone regeneration and remodeling (Kulesza et al., 2023; Calabro et al., 2019; Zondervan et al., 2021; Delany and Hankenson, 2009). The potential role of this protein in the pathogenesis of OSD is currently unknown.

In the course of Osgood-Schlatter disease, changes in the concentrations of MMP-8, MMP-13, and thrombospondin-2 are observed, which can be used to distinguish this condition from others.

Therefore, the aim of this work was to determine the potential changes in plasma concentrations and diagnostic utility of Metalloproteinase 8, Metalloproteinase 13, and Thrombospondin 2 in patients with OSD. This study is a continuation of research into changes in the plasma concentrations of selected biologically active compounds in OSD patients, with the aim of making a diagnosis, differentiating OSD patients from those with other skeletal disorders, and establishing the potential pathogenesis of OSD.

## 2 Materials and methods

The study was conducted on 140 patients (71 girls, 69 boys) who presented to the Department of Orthopedics and Traumatology of the Ludwig Zamenhoff University Children’s Clinical Hospital in Białystok with symptoms of pain in the knee area. The diagnosis of OSD was made on the basis of clinical history and X-ray images. The patients’ clinical symptoms included reported tenderness in the tibial tuberosity in ap-proximately 90% of patients, swelling (approximately 96% of patients), and pain (measured during kneeling, with 95% of patients reporting pain). The radiographs revealed an enlarged tibial tubercle contour and/or loose bone fragments at the site of the patellar ligament attachment. Tibial tuberosity fragmentation was noted in approximately 22.14% of patients. Patients included in our study had no chronic diseases, were recruited into the study immediately after being diagnosed with OSD, and had not been previously treated conservatively, including with any medications (NSAIDs).

The control group (CG) comprised 100 patients – 42 girls and 58 boys - who presented to the Department of Orthopedics and Traumatology of the Ludwig Zamenhoff University Children’s Clinical Hospital in Białystok with minor hand injuries. In addition, the individuals included in the control group did not have any chronic or infectious diseases, especially those affecting bone physiology. Patients included in the control group did not demonstrate fragmentation within the tibial tuberosity, did not complain of tenderness in the knee area, and no one reported knee pain when kneeling. Exclusion criteria included previous knee injuries and other knee conditions, especially rheumatologic conditions. Characteristics of the patients are presented in Table 1.

**TABLE 1:** Characteristics of examined groups: patients with OSD and the control group.

Feature		Patients with OSD	Control group
Number of patients		140	100
Gender		Female −71 Male −69	Female −42 Male – 58
Median age (range)		13 (11–15)	13 (12–15)
Median height (range)		160 (131–184)	159 (135–179)
Median body weight (range)		56 (30–95)	52 (32–80)
Physical activity (hours^a^)		10 (6–16)	5 (4–7)
Fragmentation of the tibial Tuberosity (%)		22.14	-
Tenderness	Yes	126	-
	No	14	100
Swelling	Yes	134	-
	No	6	100
Pain when kneeling	Yes	133	-
	No	7	100

^a^ The times given refer to the time spent on physical activity measured over a week (7 days).

The material used in the study was plasma, 4.9 mL (S-Monovette® Li-Heparin Liquid) obtained from venous blood taken from the elbow flexure, drawn on lithium heparin, which was an anticoagulant. The patients were in good condition at the time of collection. The time from the onset of the first pain symptoms to the collection of blood for the study was no longer than 1 month. The blood collected from the patients was then centrifuged at 1810 g for 10 min and placed in a freezer at −81 °C until the day the assays were performed. The concentrations of the parameters studied: MMP-8, MMP-9, and Thrombospondin 2, were measured using the immunoenzymatic ELISA method. The assay used kits from R&D Systems (MMP-8: Human MMP-8 DuoSet ELISA, Cat. no. DY206-05; MMP-13: Human DuoSet ELISA Cat. no. DY511, Minneapolis, MN, United States; Thrombospondin: Human Thrombospondin 2 ELISA Kit–Quantikine Cat. No. DTSP20, Minneapolis, MN, United States). The test procedure was carried out according to the manufacturer’s instructions included with each kit. The following sample dilutions were used: MMP-8 x20; MMP-13 × 2; Thrombospondin 2 × 4. The tests were conducted at a constant temperature of 21 °C. Both the standards and patient samples were assayed in duplicate. Plates were read at 450 nm and a correction set to 540 nm using a microplate spectrophotometer with a microplate reader, BioTek EPOCH. The precision of the kits was–MMP-8: 4.5% (intra-assay) and 7.25% (inter-assay); MMP-13: 4.9% (intra-assay) and 4.63% (inter-assay); and Thrombospondin 2: 2.6% (intra-assay) and 2.8% (inter-assay).

The study was conducted in accordance with the Declaration of Helsinki. The protocol was approved by the local Ethics Committee of the Medical University of Białystok (committee approval numbers: APK.002.580.2021, (date of approval: 16 December 2021). The study participants were minors; hence, the children were qualified to participate in the study after consulting their parents/legal guardians and obtaining their written consent.

### 2.1 Statistical analysis

Statistical analysis was performed with PQStat Software (v.1.8.4.162, Poznań, Poland. Normal distribution was determined using the Shapiro-Wilk test; the data examined had a non-parametric distribution. Further analyses were carried out using non-parametric tests: Kruskal–Wallis’s test with the Conover-Iman post hoc test for performing group comparisons, and the Spearman rank correlation test for determining correlations between parameters. Diagnostic reliability and diagnostic power of the tests were analyzed using the ROC curve. ROC curves were compared using AUC values determined by the non-parametric De Long method. Figures were created using Graph Pad Prism 5 software (GraphPad Software, La Jolla, CA, United States).

## 3 Results

### 3.1 Concentrations of MMP-8, MMP-13, and thrombospondin 2 obtained in the plasma of OSD patients and the control group

**FIGURE 1:**
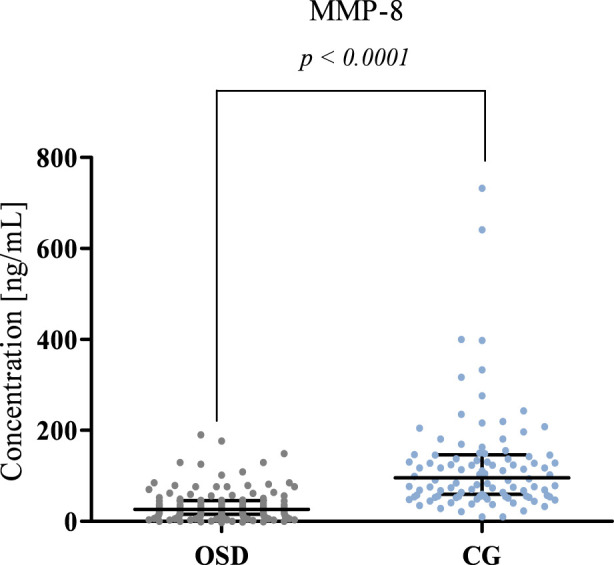
MMP-8 concentration (with marked median and interquartile range) obtained in the plasma of subjects with Osgood-Schlatter disease (OSD) and in the control group (CG). MMP-8 concentrations were obtained by ELISA method.

**FIGURE 2:**
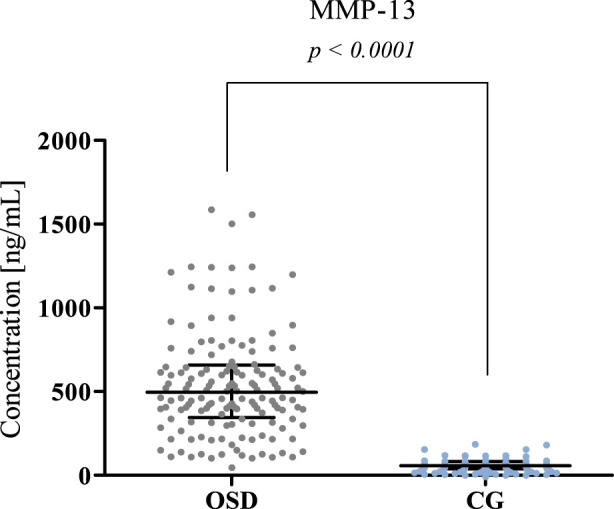
MMP-13 plasma concentration in patients with Osgood-Schlatter disease (OSD) and in the control group (CG) with median and interquartile range indicated. MMP-13 concentrations were obtained by ELISA method.

**FIGURE 3:**
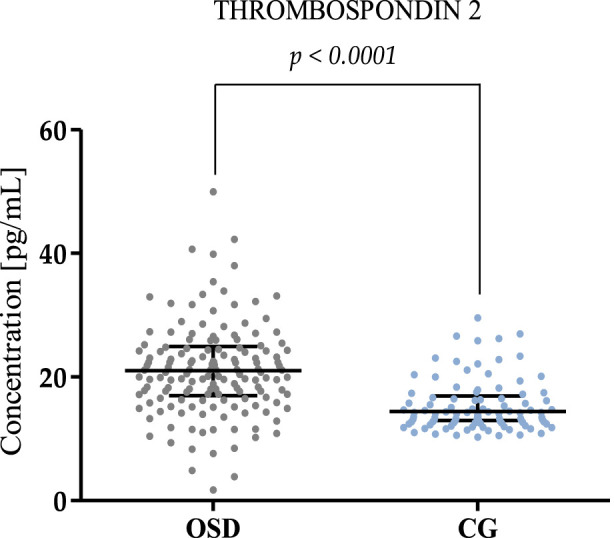
Plasma Thrombospondin 2 concentration in patients with Osgood-Schlatter disease (OSD) and in the control group (CG) (with median and interquartile range). Thrombospondin 2 concentrations were obtained by ELISA method.

The results obtained for MMP-8, MMP-13, and Thrombospondin 2 concentrations in the OSD patients and the control group (CG) are presented in Table 2 and Figures 1–3.

**TABLE 2:** Plasma concentrations of the studied parameters–MMP-8, MMP-13, Thrombospondin 2 obtained in patients with OSD and in the control group.

Patients with OSD			
	MMP-8 [ng/mL]	MMP-13 [ng/mL]	Thrombospondin 2 [pg/mL]
Median	26.60	496.5	21.05
Min – max range	3.01–195.8	58.98–1,600	2.148–50.37
IQR	15.51–45.36	346.1–658.6	16.96–24.93
Mean ± SD	37.25 ± 2.74	548.10 ± 25.40	21.4 ± 0.60
Control group			
Median	95.91	58.08	14.43
Min – max range	14.79–737.5	6.128–199.40	10.68–29.99
IQR	59.63–146.60	38.83–82.56	12.98–16.90
Mean ± SD	125.9 ± 11.14	64.56 ± 3.82	15.59 ± 0.41

#### 3.1.1 MMP-8 concentration

Significantly lower plasma levels of MMP-8 were observed in OSD patients (median: 26.60 ng/mL) compared to the concentration values obtained in the control group (median: 95.91 ng/mL, p < 0.0001). The results obtained are presented in Figure 1.

#### 3.1.2 MMP-13 concentration

Significantly higher MMP-13 concentrations were obtained in the OSD group (median: 496.5 ng/mL) compared to the concentrations obtained in the CG (median: 58.08 ng/mL, p < 0.0001). The results obtained are presented in Figure 2.

#### 3.1.3 Thrombospondin 2 concentration

Similarly to MMP-13, higher concentrations of Thrombospondin 2 were found in the OSD group (median: 21.05 pg/mL) compared to the concentration values obtained in the CG group (median: 14.43 pg/mL, p < 0.001). The results obtained are presented in Figure 3.

### 3.2 Evaluation of correlation using Spearman’s method

Correlation evaluation of MMP-8 and MMP-13 and Thrombospondin 2 is shown in Table 3. Spearman’s non-parametric test was selected for the analysis.

**TABLE 3:** Evaluation of correlations between studied parameters: MMP-8, MMP-13, and Thrombospondin 2 in patients with OSD.

Tested correlations			r	p
MMP-8	vs	MMP-13	−0.5781	**<0.001** ^a^
MMP-8	vs	Thrombospondin 2	−0.3391	**<0.001** ^a^
MMP-13	vs	Thrombospondin 2	0.3937	**<0.001** ^a^

^a^ —bold text indicates statistically significant results.

**FIGURE 4:**
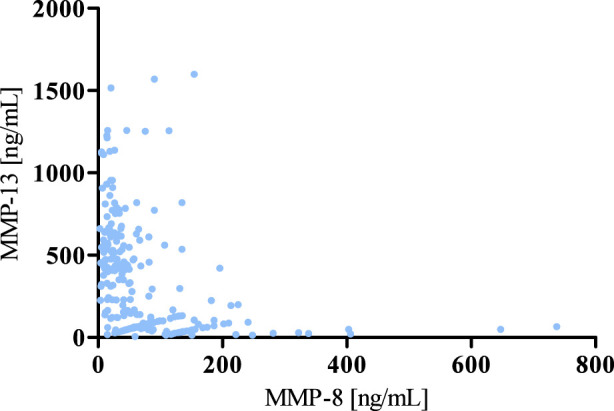
Spearman’s negative correlation between MMP-8 and MMP-13.

**FIGURE 5:**
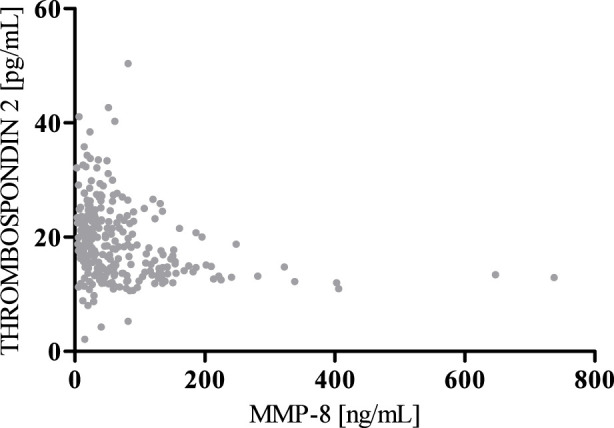
Spearman’s negative correlation between MMP-8 and Thrombospondin 2.

**FIGURE 6:**
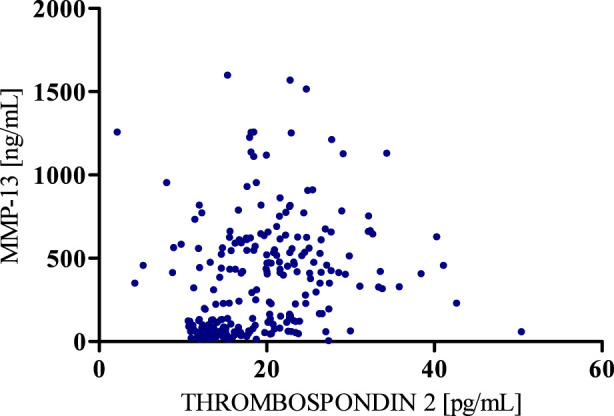
Spearman’s positive correlation between MMP-13 and Thrombospondin 2.

According to the results of correlation analysis, statistically significant negative correlations were observed between MMP-8 and MMP-13 (r = −0.5781; p < 0.001) as well as between MMP-8 and Thrombospondin 2 (r = −0.3391; p < 0.001). We also found a statistically significant positive correlation between MMP-13 and Thrombospondin 2 (r = 0.3937; p < 0.001) (Figures 4–6).

### 3.3 Diagnostic criteria of MMP-8, MMP-13, and thrombospondin 2

The diagnostic criteria are as follows: diagnostic sensitivity (SE), diagnostic specificity (SP), positive predictive value (PPV), negative predictive value (NPV), and area under curve (AUC) of MMP-8, MMP-13, and Thrombospondin 2; OSD results are presented in Table 4.

**TABLE 4:** Values of diagnostic criteria (SE, SP, PPV, NPV, AUC) obtained for the tested MMPs and Thrombospondin 2.

Parameter	SE [%]	SP [%]	PPV [%]	NPV [%]	AUC
MMP-8	76.67	92.87	54.55	93.71	0.889
MMP-13	83.33	90.46	62.50	91.14	0.989
Thrombospondin 2	62.93	81.34	53.13	93.15	0.776

The highest SE value among all tested parameters was obtained for MMP-13 (83.33%). A slightly lower SE value was observed for MMP-8 (76.67%), while the lowest value among the tested parameters was obtained for Thrombospondin 2 (62.93%).

In the case of SP, the highest value of this parameter, amounting to 92.87%, was observed for MMP-8. A slightly lower value of 90.46% was observed for MMP-13. The lowest SP value was obtained for Thrombospondin 2%–81.34%.

For PPV, the highest value was obtained for MMP-13, and it amounted to 62.50%. PPV values obtained for MMP-8 and Thrombospondin 2 were similar and amounted to 54.55% and 53.13%, respectively.

The NPV values obtained for the tested parameters MMP-8, MMP-13, and Thrombospondin 2 were similar and amounted to 93.71%, 91.14%, and 93.15%, respectively.

**FIGURE 7:**
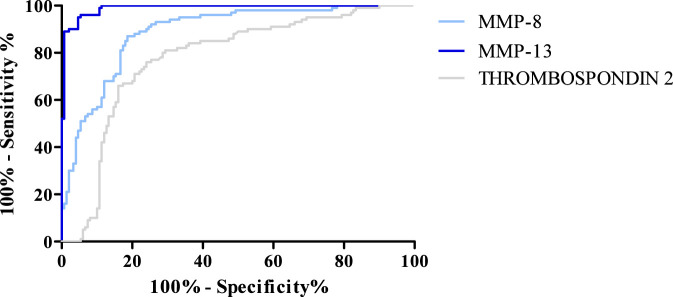
ROC curve for MMP-8, MMP-13, and Thrombospondin 2.

The AUC value was also determined for MMP-8, MMP-13, and Thrombospondin 2. An AUC value of 0.5 is the borderline of the diagnostic usefulness of the test. The highest AUC was obtained for MMP-13 (0.989). Lower, but still high, results were obtained for MMP-8 (0.889) and Thrombospondin 2 (0.776). The AUC values obtained for all parameters are presented in Figure 7.

## 4 Discussion

Osgood-Schlatter disease (OSD) has a relatively high incidence. It is estimated to affect one in ten athletically active people, especially during adolescence. Although it is self-limiting in most cases, it forces active people to give up physical exercise. Some-times it may also require surgical intervention, which translates into longer rehabilitation time and the risk of potential complications after surgery (Guszczyn et al., 2024; Ladenhauf et al., 2020; Schultz et al., 2022).

The exact pathomechanism of OSD is currently unknown, and it has been postulated that risk factors such as BMI and pathologies in the muscles and patellar mechanics are involved (Guszczyn et al., 2023; Smith and Varacallo, 2025). The involvement of biologically active compounds, such as matrix metalloproteinases (MMPs) or other compounds, is also not to be ruled out. In previous studies, our team showed that the concentration of particular MMPs decreased or increased in the plasma of patients with OSD. This may indicate their involvement in the pathogenesis of this condition (Kulesza et al., 2023). The aim of this study was therefore to investigate changes in the concentration of selected MMPs from the collagenase group: MMP-8 and MMP-13, as well as changes in the concentration of Thrombospondin 2 in plasma in patients with OSD compared to patients with knee injuries without OSD.

The first compound studied in our work was MMP-8. As mentioned above, this enzyme belongs to the collagenase group. It is expressed in human fibroblasts (including synovial membrane fibroblasts), chondrocytes, macrophages, myocytes, osteoblasts, and osteoclasts (Baidya et al., 2024; Steinl et al., 2013; Larrouture et al., 2021). The most important role of MMP-8 is related to the stimulation of neutrophil migration and, consequently, the induction of inflammation (Dejonckheere et al., 2011). In the course of OSD, there is inflammation of the patellar tendon attachment on the tibial tuberosity (Guszczyn et al., 2023). In our study, we have shown that patients with OSD have statistically lower MMP-8 levels (median: 26.60 ng/mL) compared to the control group (median: 95.91 ng/mL; p < 0.0001). We are currently the first research team to perform a similar experiment, therefore, we are not able to compare our results with similar studies. However, it should be noted that in our team’s previous work, we also found a decrease in the concentrations of selected MMPs in patients with OSD (Kulesza et al., 2024). For example, MMP-8 concentrations were lower (median: 0.24 ng/mL) in OSD patients compared to controls (median: 1.02 ng/mL; p < 0.0001). The lower levels of MMP-8 in OSD patients may indicate that this enzyme is involved in the development of inflammation, including in patients with other knee injuries (control group). Due to the high heterogeneity of the control group, further studies focusing on changes in MMP-8 levels in patients with OSD and other knee injuries should be conducted. Nevertheless, due to the lower concentrations of MMP-8 in patients with OSD compared to those with other knee injuries, one can postulate the usefulness of this enzyme as an additional test in the diagnosis of this condition. Moreover, in the future, changes in the concentrations of this enzyme may serve as a prognostic factor indicating the need for surgery. Again, however, this requires further research.

MMP-13 also belongs to the collagenase group. This enzyme is highly expressed in osteoblasts, osteocytes, chondrocytes, and myocytes (Ponte et al., 2022; Pivetta et al., 2011); it has been shown to play an important role in bone physiology, where it is involved in the increased activity of osteoclasts and thus affects bone resorption, contributing to the destruction of bone elements (Kulesza et al., 2023; Augustina et al., 2023). According to our results, OSD patients have significantly higher levels of MMP-13 (median: 496.5 ng/mL) compared to the control group (median: 58.08 ng/mL; p < 0.0001). This may suggest that this enzyme is involved in tissue destruction in OSD. However, we are unable to compare our results with those obtained in other studies, as no one has investigated the concentration of this parameter in OSD yet. Nevertheless, our research is partly consistent with the research on other diseases, such as osteoarthritis. In a study by Aripaka et al. (Aripaka et al., 2022), patients with osteoarthritis had higher expression of MMP-13, and the level of this expression increased at higher stages of the disease (Aripaka et al., 2022). MMP-13 imbalance is also observed in the course of another bone disease, arthritis. Patients with this disease have higher serum concentrations of MMP-13 than healthy patients (Xin et al., 2021; Hu and Ecker, 2021). This indicates that this enzyme is involved in bone degradation and that its concentration in blood is high enough to exceed the detection threshold and can be considered as a target in the treatment and diagnosis of osteoskeletal diseases, including OSD. This applies, for example, to the topical use of synthetic MMPs inhibitors (Liu and Khalil, 2017).

Thrombospondin 2 is an extracellular matrix protein. This protein has been shown to play an important role in numerous processes related to bone physiology. Thrombospondin 2 is involved in bone collagen synthesis and bone tissue mineralization. It also inhibits bone angiogenesis and mesenchymal cell proliferation. However, the most important role of this protein is related to bone healing (Zondervan et al., 2021; Chen et al., 2024). In our study, we found higher levels of Thrombospondin 2 in subjects with OSD (21.05 pg/mL median: 14.43 pg/mL, p < 0.001) compared to subjects with other knee injuries (median: 14.43 pg/mL, p < 0.001). This result may indicate that the ongoing bone healing process is more pronounced in OSD patients, possibly due to the area of tissue involved in the injury. Unfortunately, we are unable to refer to other results concerning the activity of Thrombospondin 2 in the musculoskeletal system, as most studies on its role are currently being conducted on animals, while its usefulness as a marker has been confirmed in patients with liver disease (Okumura et al., 2024). However, the results of these studies indicate that Thrombospondin 2 is important both as a protein responsible for bone healing and as a potential biomarker measured in peripheral blood. Our study confirms the initial potential of this protein as an additional test in OSD diagnosis and also suggests that this protein is involved in the healing of bone structures in the course of this condition, as its concentration increases above the threshold of detection.

It is unfortunate that our work has some limitations. First, we used only one type of research methodology in the assays, which is the ELISA method. In the future, we will also repeat this research using other assay methods. Second, we did not take into account the influence of several biological factors, i.e., BMI, or the effect of puberty on changes in the concentration of the studied parameters. Moreover, the study group was heterogeneous, therefore we would try to standardize the study group in the future. Finally, the study we conducted was a single-center study, so it could also be repeated in another research unit.

## 5 Clinical implications of the findings

Changes in MMP-13 and Thrombospondin 2 levels have several beneficial clinical implications. Although OSD is a self-limiting disease, the duration of symptoms is relatively long (Chandra et al., 2024; Preuss et al., 2024). OSD primarily affects young athletes, who are forced to stop intensive training due to pain, which can lead to their complete elimination from sports early in their athletic careers (Guszczyn et al., 2024). Such situations can have a very negative impact on the mental health of children and adolescents with OSD (Guszczyn et al., 2024). For this reason, it would be worthwhile to find a research method that would allow doctors to monitor the course of the disease and thus assess the effectiveness of the treatment provided. Monitoring the course of the disease with MMP-13, and Thrombospondin 2 is highly promising in this situation. Changes in the concentrations of these compounds could probably be used in the future to assess the effectiveness of treatment and the potential necessity of modifying treatment. Concentrations of MMP-13 and Thrombospondin 2 probably return to baseline values after the healing process is complete, and therefore have high potential in assessing patient recovery. Performing assays of these two compounds would allow us to assess whether the healing process has ended or whether the patient requires further treatment. However, these are only assumptions and speculations that need to be supported by studies on a larger number of patients.

In conclusion, our results suggest that MMP-13 is involved in the pathogenesis of OSD, while Thrombospondin 2 may be associated with bone healing in the course of this condition and indicate their potential as markers helpful in the diagnosis of this disease.

## 6 Conclusion

In conclusion, MMP-13 and MMP-8 may be involved in the pathogenesis of OSD, while Thrombospondin 2 is involved in healing in the course of the disease. MMP-13 and Thrombospondin 2 can potentially be used as additional tests in differentiating OSD from other knee conditions. However, their unequivocal identification as diagnostic and differential parameters requires further study.

## Data Availability

The raw data supporting the conclusions of this article will be made available by the authors, without undue reservation.
